# Exploring the Resurgence of a Neglected Disease: Lessons From the 2023–2024 Mpox Outbreak in Rio de Janeiro, Brazil

**DOI:** 10.1093/cid/ciae290

**Published:** 2024-07-16

**Authors:** Mayara Secco Torres Silva, Carolina Coutinho, Thiago Silva Torres, Monica Avelar Magalhães, Carolyn Yanavich, Amanda Echeverría-Guevara, Matheus Oliveira Bastos, Pedro Silva Martins, Maira Braga Mesquita, Paula Pereira de Souza Reges, Maria Roberta Meneguetti, Ana Paula Lovetro Santana, Marcela Terra, Estevão Portela Nunes, Flavia Cristina Serrão Lessa, Ronaldo Ismério Moreira, Eduardo Mesquita Peixoto, Karolyne Wolch de Almeida Paulo, Andryelle Cristina Sant’Ana, Edson Elias da Silva, Sandra Wagner Cardoso, Valdilea Gonçalves Veloso, Beatriz Grinsztejn, for the Instituto Nacional de Infectologia Evandro Chagas (INI)–Fiocruz Mpox Study Group, João Paulo Bortot Soares, Giselle Hottz, Cicero Santos, Bruna Fabris Rendelli, Luciana Patricia Tuccori, Jadir Rodrigues Fagundes Neto, Lucilene Freitas, Mayumi Wakimoto, Katia Azevedo, Rodrigo Otavio da Silva Escada, Maria Pia Diniz Ribeiro, Isabel Cristina Ferreira Tavares, Desirée Vieira Santos, Luiz Ricardo Siqueira Camacho, Leonardo Rosadas, Luisa Fares, Pedro Amparo, Hugo Boechat Andrade, José Ricardo Hildebrant Coutinho, Hugo Perazzo Pedroso Barbosa, Sandro Nazer, Guilherme Amaral Calvet, Rodrigo Caldas Menezes, Sandro Antônio Pereira

**Affiliations:** Instituto Nacional de Infectologia Evandro Chagas, Fiocruz, Rio de Janeiro, Brazil; Instituto Nacional de Infectologia Evandro Chagas, Fiocruz, Rio de Janeiro, Brazil; Instituto Nacional de Infectologia Evandro Chagas, Fiocruz, Rio de Janeiro, Brazil; Instituto de Comunicação e Informação Científica e Tecnológica em Saúde, Fiocruz, Rio de Janeiro, Brazil; Instituto Nacional de Infectologia Evandro Chagas, Fiocruz, Rio de Janeiro, Brazil; Instituto Nacional de Infectologia Evandro Chagas, Fiocruz, Rio de Janeiro, Brazil; Instituto Nacional de Infectologia Evandro Chagas, Fiocruz, Rio de Janeiro, Brazil; Instituto Nacional de Infectologia Evandro Chagas, Fiocruz, Rio de Janeiro, Brazil; Instituto Nacional de Infectologia Evandro Chagas, Fiocruz, Rio de Janeiro, Brazil; Instituto Nacional de Infectologia Evandro Chagas, Fiocruz, Rio de Janeiro, Brazil; Instituto Nacional de Infectologia Evandro Chagas, Fiocruz, Rio de Janeiro, Brazil; Instituto Nacional de Infectologia Evandro Chagas, Fiocruz, Rio de Janeiro, Brazil; Instituto Nacional de Infectologia Evandro Chagas, Fiocruz, Rio de Janeiro, Brazil; Instituto Nacional de Infectologia Evandro Chagas, Fiocruz, Rio de Janeiro, Brazil; Instituto Nacional de Infectologia Evandro Chagas, Fiocruz, Rio de Janeiro, Brazil; Instituto Nacional de Infectologia Evandro Chagas, Fiocruz, Rio de Janeiro, Brazil; Instituto Nacional de Infectologia Evandro Chagas, Fiocruz, Rio de Janeiro, Brazil; Instituto Oswaldo Cruz, Fiocruz, Rio de Janeiro, Brazil; Instituto Oswaldo Cruz, Fiocruz, Rio de Janeiro, Brazil; Instituto Oswaldo Cruz, Fiocruz, Rio de Janeiro, Brazil; Instituto Nacional de Infectologia Evandro Chagas, Fiocruz, Rio de Janeiro, Brazil; Instituto Nacional de Infectologia Evandro Chagas, Fiocruz, Rio de Janeiro, Brazil; Instituto Nacional de Infectologia Evandro Chagas, Fiocruz, Rio de Janeiro, Brazil

**Keywords:** mpox, Brazil, men who have sex with men, sexually transmitted infections, Latin America

## Abstract

Following the 2022 global mpox outbreak, diagnoses decreased worldwide, even in settings with limited vaccine access. In 2023–2024, a new outbreak emerged in Rio de Janeiro, Brazil, highlighting the importance of continuous surveillance, preventive measures such as vaccination in vulnerable populations, and treatment options, emphasizing equitable global health technology distribution.

## Graphical Abstract

**Graphical Abstract:**
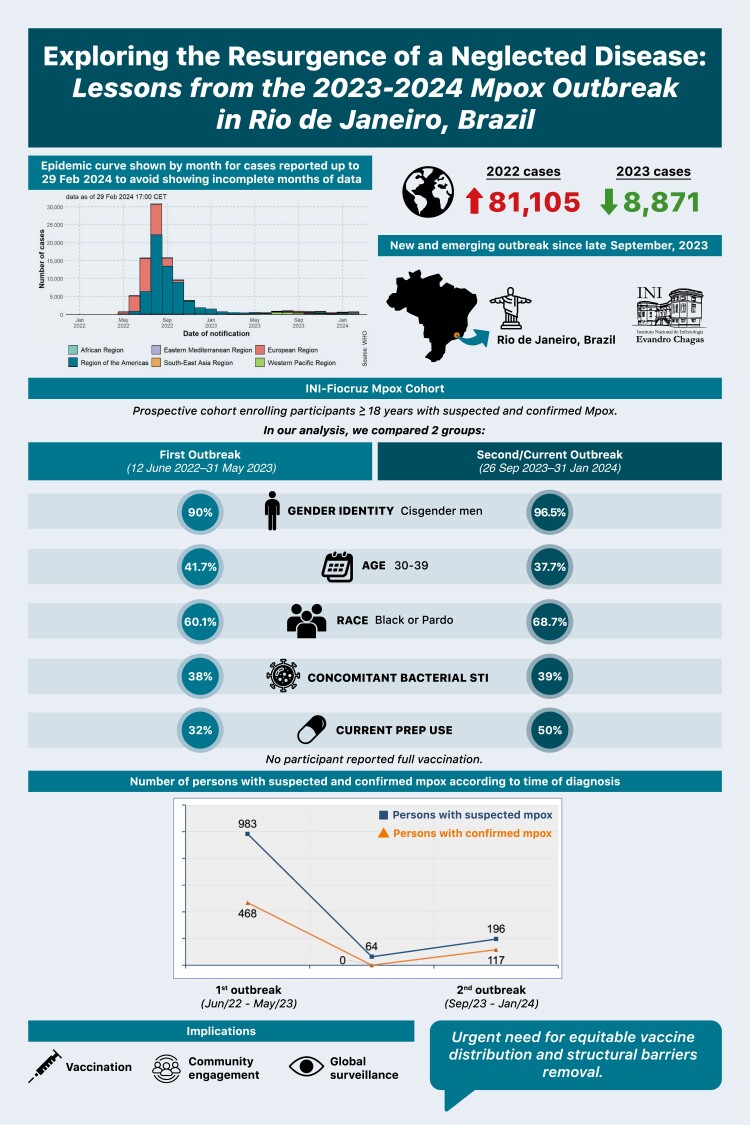

**(See the Editorial Commentary by Telford et al. on pages 660–2.)**

After a peak in July–August 2022, global mpox diagnoses progressively decreased. While 81 105 cases were reported globally during the outbreak, only 8871 persons with mpox were identified in 2023, meaning an approximate 90% reduction [1]. Various hypotheses, such as vaccination programs, community engagement, or cyclic virus behavior, attempt to explain the global decline. Despite social inequities and regional disparities in vaccine supply, even countries without mpox vaccines saw a steady drop in cases, contrasting with outbreaks in the West-Pacific and African regions in mid-2023 [1]. The 2022 mpox multinational outbreak significantly affected the Americas, with Brazil recording 10 967 confirmed diagnoses by 31 December 2023.

In May 2023, the World Health Organization declared the end of the mpox emergency, emphasizing the importance of sustained efforts for disease control. This decision had an impact on national mpox surveillance efforts, particularly in Brazil, where the last national data update was in August 2023 [2]. In Rio de Janeiro, the third-largest state in the country and one of the epicenters of the first mpox outbreak, 1468 individuals were diagnosed with mpox, with 95% of cases reported until 31 May 2023. Between 1 June 2023 and 25 September 2023, only 2 isolated cases were reported, showing no sign of sustained transmission [3]. Herein, we present initial evidence of an emerging and ongoing mpox outbreak in Rio de Janeiro, Brazil, initiated in late September 2023. We also compared the characteristics of participants diagnosed with mpox during this new outbreak (26 September 2023 to 31 January 2024) with those diagnosed in the first outbreak (12 June 2022 to 31 May 2023).

## METHODS

This is a single-center, prospective cohort study enrolling participants aged 18 years or older with suspected mpox from 12 June 2022 to 31 January 2024, followed up at the Evandro Chagas National Institute of Infectious Diseases (INI-Fiocruz), the major infectious diseases referral center in Rio de Janeiro. Participants were diagnosed with mpox if they yielded a positive monkeypoxvirus (MPXV) polymerase chain reaction result from any swab specimens. All cohort participants are offered multisite mpox testing (oropharyngeal, rectal, and lesion swabs) and other sexually transmitted infections (STIs) and viral hepatitis testing, including rapid *Treponema pallidum* test followed by nontreponemal test (Venereal Disease Research Laboratory [VDRL]) for confirmation purposes, rectal swabs for chlamydia and gonorrhea (Abbott Real Time platform), and hepatitis B surface antibody and anti–hepatitis C virus (HCV) rapid tests. INI-Fiocruz Mpox cohort procedures and initial results are described elsewhere [4]. For this analysis, we included only participants with confirmed mpox and compared their sociodemographic, behavioral, clinical, and laboratory characteristics according to period of enrollment: (1) first outbreak (12 June 2022 to 31 May 2023) and (2) second/current outbreak (26 September 2023 to 31 January 2024). We used χ^2^ or Fisher exact tests to compare qualitative variables and Wilcoxon rank-sum test for quantitative variables. This study was approved by the Ethics Review Board at INI-Fiocruz (CAAE#61290422.0.0000.5262). Participants provided written informed consent.

## RESULTS

During the first outbreak (12 June 2022 to 31 May 2023), of 983 participants with suspected mpox, 468 (47.6%) were confirmed. Between outbreaks (1 June 2023 to 25 September 2023), we tested 64 individuals with suspected mpox, with no confirmed cases. However, on 26 September 2023, the first participant with mpox in almost 4 months was identified. There was a steady and consistent increase in mpox diagnoses from 26 September 2023 to 31 January 2024, with 196 participants assessed, of whom 117 (59.7%) confirmed mpox, indicating a second mpox outbreak in Rio de Janeiro. None of the participants reported being fully vaccinated, and 1 participant with confirmed mpox had received only 1 dose of the JYNNEOS vaccine. We had 3 potential reinfection cases that could not be confirmed by phylogenetical analysis until this publication.

Overall, the second/current mpox outbreak mostly affected individuals aged 30–39 years (n = 43/117 [37.7%]), cisgender men (n = 113/117 [96.5%]), men who have sex with men (n = 110/113 [97.3%]), those who self-identified as Black/*Pardo* (n = 79/115 [68.7%]), and those reporting post-secondary education as the highest level of schooling (n = 70/116 [60.3%]) (Table 1). Compared to the first outbreak, the second/current outbreak presented higher proportions of cisgender men (96.5% vs 90.0%, *P* = .01), as well as men who reported sex with men (97.3% vs 91.0%, *P* = .04), >1 sex partner (69.0% vs 53.7%, *P* < .01), and anal sex (88.8% vs 68.4%, *P* < .01) in the 30 days prior to symptom onset. Human immunodeficiency virus (HIV) coinfection was more frequent in the second/current outbreak (62.0% vs 51.2%, *P* = .05), as well as current preexposure prophylaxis (PrEP) use at enrollment (50.0% vs 32.0%, *P* = .01). Participants took more time from symptom onset to first assessment at INI-Fiocruz during the second/current outbreak (>5 days: 71.0% vs 59.5%, *P* = .02). Age, race, educational level, and clinical characteristics, including concurrent STIs and HCV past/current infection, were similar across the outbreaks.

**Table 1.** Sociodemographic, Behavioral, and Clinical Characteristics of Participants Diagnosed With Mpox, According to Outbreak (N = 551)

Characteristic	First Outbreak (June 2022–May 2023) (N = 468)	Second Outbreak (September 2023–January 2024) (N = 117)	*P* Value^a^
Age, y			
Median (IQR)	33 (28–40)	32 (28–40)	.30
18–24	54/468 (11.5)	19/117 (16.3)	.50
25–29	95/468 (20.3)	25/117 (21.4)	
30–39	195/468 (41.7)	43/117 (37.7)	
≥40	124/468 (26.5)	30/117 (25.6)	
Gender identity			.**01**
Cisgender men	421/468 (90.0)	113/117 (96.5)	
Cisgender women	33/468 (7.1)	3/117 (2.6)	
Nonbinary	0/468	1/117 (0.9)	
*Travesti* or transgender women	14/468 (2.9)	0/117	
Transgender men	0/468	0/117	
Men who have sex with men	364/400 (91.0)	110/113 (97.3)	.**04**
Race			.20
White	152/389 (39.1)	35/115 (30.4)	
Indigenous	3/389 (0.8)	1/115 (0.9)	
Black or *Pardo*	234/389 (60.1)	79/115 (68.7)	
Educational level			.60
Primary	32/399 (8.0)	6/116 (5.2)	
Secondary	135/399 (33.9)	40/116 (34.5)	
Postsecondary	232/399 (58.1)	70/116 (60.3)	
Time from symptom onset to first medical assessment at INI-Fiocruz			.**02**
Median, d (IQR)	6 (4–10)	7 (5–12)	
≤5 d	176/435 (40.5)	33/115 (29.0)	.**02**
>5 d	259/435 (59.5)	82/115 (71.0)	
Reported sex contact^b^	390/437 (89.2)	108/115 (94.0)	.13
No. of sex partners^b^			**<**.**01**
≤1	148/320 (46.3)	34/108 (31.0)	
>1	172/320 (53.7)	74/108 (69.0)	
Reported anal sex^b^	195/285 (68.4)	103/116 (88.8)	**<**.**01**
Sexual contact with a potential mpox case^b^	85/400 (21.3)	20/115 (17.0)	.40
People with HIV	234/457 (51.2)	72/117 (62.0)	.**05**
HIV RNA >200 copies/mL	33/234 (14.1)	12/72 (16.7)	.50
CD4^+^ T-lymphocytes <350 cells/μL	27/207 (13.0)	8/69 (11.6)	.30
Current PrEP use	73/228 (32.0)	22/44 (50.0)	.**01**
Active syphilis^c^	93/431 (21.6)	30/116 (25.9)	.30
Anorectal gonorrheae	32/385 (8.3)	14/110 (13.0)	.20
Anorectal chlamydia	34/385 (8.8)	13/110 (12.0)	.30
Concomitant bacterial STI^d^	146/384 (38.0)	43/111 (39.0)	.76
Hepatitis B	6/420 (1.4)	2/116 (1.7)	.70
Hepatitis C	26/430 (6.0)	89/114 (9.5)	.20
Any systemic signs or symptoms^e^	380/445 (85.4)	66/83 (78.1)	.06
Ophthalmological complaints	26/451 (5.7)	3/116 (2.6)	.20
Pharyngitis and/or odynophagia	121/454 (26.7)	24/116 (20.7)	.20
Proctitis	105/464 (22.6)	37/116 (31.7)	.**04**
Hospitalization during follow-up	49/468 (10.5)	10/117 (8.6)	.50

Data are presented as no./No. (%) unless otherwise indicated.

Bold values as *P* <.05.

Abbreviations: HIV, human immunodeficiency virus; INI, Instituto Nacional de Infectologia Evandro Chagas; IQR, interquartile range; PrEP, preexposure prophylaxis; STI, sexually transmitted infection.

^a^Fisher exact test; Wilcoxon rank-sum test; χ^2^ test.

^b^In the 30 days before symptom onset.

^c^Active syphilis defined as Venereal Disease Research Laboratory titer ≥1:8.

^d^Including active syphilis, anorectal chlamydia, and/or gonorrheae.

^e^Including fever, myalgia, arthralgia, asthenia, and/or headache.

Among people with HIV (PWH) in the second/current outbreak (n = 72), 12 (16.7%) had an HIV RNA load >200 copies/mL and 8 (11.6%) had related immunosuppression (CD4^+^ T-lymphocyte count <350 cells/μL). Furthermore, 6 (8.3%) were diagnosed with concomitant opportunistic infections (Kaposi sarcoma, n = 3; tuberculosis, n = 2; cryptococcosis, n = 1). Three (5.8%) participants had suspected mpox-associated immune reconstitution inflammatory syndrome, displaying worsening mucocutaneous lesions after reinitiating antiretroviral therapy (ART). Comparing both outbreaks, no differences were observed related to ART adherence, HIV RNA load, or CD4^+^ T-lymphocyte count.

The hospitalization rate during the second/current outbreak was 8.6%, similar to the first outbreak (10.5%, *P* = .50), and from 26 September 2023 to 31 January 2024, no deaths related to mpox were reported.

## DISCUSSION

This marks the inaugural report of a new mpox outbreak in Rio de Janeiro, Brazil, following a period with no signs of sustained transmission. Once again, it disproportionally impacts gender-diverse and sexually diverse populations, PWH, and Black/*Pardo* individuals. Similar to the first outbreak, sexual transmission prevails in this new outbreak, continuing to heavily affect these groups. However, there is a potential for changing over time, as illustrated by data from the first outbreak showing higher proportion of mpox among women in the later months [5]. Of note, no cases were reported among *travestis* or transgender women during the second/current outbreak, which might also change over time as supported by previous data [5].

Our findings suggest a continuous, unnoticed transition of mpox in Rio de Janeiro, Brazil. These changes might be linked to unmeasured variables, such as changes in sexual behavior, decreased level of clinical suspicion, and the occurrence of oligosymptomatic cases, contributing to mpox underdiagnosis [6–9]. A plausible hypothesis suggests that undetected and low-level circulation of MPXV in Rio de Janeiro, Brazil, between June and September 2023, might have precipitated the current outbreak due to waning immunity and the absence of vaccination strategies. Another hypothesis proposes the persistent carriage of mpox in certain individuals, notably among PWH with severe immunosuppression, albeit representing a small fraction of the total diagnosed cases in both outbreaks. The recent discovery of MPXV detection in wastewater, even in a setting with a low prevalence of mpox, further emphasizes the first hypothesis [10].

Of note, the higher proportion of participants enrolled in HIV care or PrEP services during the second/current outbreak is likely a result of their preexisting engagement, facilitating prompt identification of new cases. Even though a single-center study has limited generalizability, INI-Fiocruz followed up 30% of persons with mpox in Rio de Janeiro State during the first outbreak, currently being responsible for up to 85% of total individuals with mpox in the state [3]. This is likely due to its extensive work in the field of HIV care and prevention, with our mpox cohort actively enrolling participants with suspected mpox throughout the year even in the context of case decline. Similarly, INI-Fiocruz has been actively enrolling participants in a randomized clinical trial evaluating tecovirimat for the treatment mpox since March 2023. This trial represents only current option to access tecovirimat in Brazil, which may also explain the concentration of cases in our center [11].

Our findings underscore the critical need to enhance ongoing surveillance strategies to detect emerging STIs within the framework of HIV care and prevention services. Swift identification and implementation of preventive measures for conditions like mpox may effectively prevent outbreak resurgence. As community transmission advances, PWH who are not actively engaged in HIV care, including late presenters and those on irregular ART, face heightened susceptibility to severe courses of mpox disease, as evidenced previously. In addition to collaborating with community stakeholders to devise preventive strategies, the implementation of routine mpox vaccination in low- or middle-income countries is of significant relevance, especially targeting the most affected populations. Ultimately, this ongoing clade II 2023–2024 mpox outbreak in Brazil serves as a stark reminder of the imperative to overcome the structural barriers relegating mpox to a neglected disease status, which has contributed to its resurgence in countries across the Global South, as exemplified by a clade I outbreak in Congo, also driven by sexual transmission [12]. Notably, none of these countries had access to mpox vaccination. This underscores the urgent need for concerted global efforts to control mpox, ensuring equitable distribution of healthcare technologies and addressing the structural determinants that still impact HIV continuum of care outcomes worldwide.

## Notes

***Acknowledgments*.** The authors thank all study participants, as well as the INI-Fiocruz inpatient unit's assistant team. The authors also acknowledge the INI-Fiocruz Mpox Study Group: João Paulo Bortot Soares, Giselle Hottz, Cicero Santos, Bruna Fabris Rendelli, Luciana Patricia Tuccori, Jadir Rodrigues Fagundes Neto, Lucilene Freitas, Mayumi Wakimoto, Katia Azevedo, Rodrigo Otavio da Silva Escada, Maria Pia Diniz Ribeiro, Isabel Cristina Ferreira Tavares, Desirée Vieira Santos, Luiz Ricardo Siqueira Camacho, Leonardo Rosadas, Luisa Fares, Pedro Amparo, Hugo Boechat Andrade, José Ricardo Hildebrant Coutinho, Hugo Perazzo Pedroso Barbosa, Sandro Nazer, Guilherme Amaral Calvet, Rodrigo Caldas Menezes, and Sandro Antônio Pereira.

***Financial support*.** This work was supported by the Instituto Nacional de Infectologia Evandro Chagas, Fundação Oswaldo Cruz and the Brazilian Ministry of Health. TST and BG were partially funded by the Conselho Nacional de Desenvolvimento Científico e Tecnológico (CNPq) and Fundação de Amparo à Pesquisa do Estado do Rio de Janeiro (FAPERJ).

## References

[1] Centers for Disease Control and Prevention . 2022–2023 monkeypox outbreak global map. 2023. Available at: https://www.cdc.gov/poxvirus/monkeypox/response/2022/world-map.html. Accessed 12 January 2024.

[2] Ministério da Saúde . Mpox: Atualização dos casos. 2023. Available at: https://www.gov.br/saude/pt-br/composicao/svsa/resposta-a-emergencias/coes/monkeypox/atualizacao-dos-casos. Accessed 16 January 2024.

[3] Coordenação de Informação Estratégica em Vigilância em Saúde do Rio de Janeiro (CIEVS-RJ) . Mpox. 2024. Available at: https://lookerstudio.google.com/reporting/bd212168-2f5b-49f3-b4e1-8529ffbc570a/page/p_55jso4e1wc. Accessed 12 January 2024.

[4] Silva MST , Coutinho C, Torres TS, et al Ambulatory and hospitalized patients with suspected and confirmed mpox: an observational cohort study from Brazil. Lancet Reg Health Am 2022; 17:100406. 36776570 10.1016/j.lana.2022.100406 PMC9904017

[5] Silva M , Torres T, Coutinho C, et al The epidemiological profile of mpox cases in Rio de Janeiro, Brazil: changes over time during the 2022 outbreak. In: 12th IAS Conference on HIV Science. Brisbane, Australia, 2023.

[6] Sahra S , Villalobos RO, Scott BM, et al The diagnostic dilemma for atypical presentation of progressive human mpox. BMC Infect Dis 2023; 23:850. 38053027 10.1186/s12879-023-08852-2 PMC10696784

[7] De Baetselier I , Van Dijck C, Kenyon C, et al Retrospective detection of asymptomatic monkeypox virus infections among male sexual health clinic attendees in Belgium. Nat Med 2022; 28:2288–92. 35961373 10.1038/s41591-022-02004-w PMC9671802

[8] Van Dijck C , Hens N, Kenyon C, Tsoumanis A. The roles of unrecognized monkeypox cases, contact isolation and vaccination in determining epidemic size in Belgium. A modelling study. Clin Infect Dis 2022;76:e1421–3. 10.1093/cid/ciac723 36052546

[9] Torres TS , Silva MST, Coutinho C, et al Evaluation of mpox knowledge, stigma, and willingness to vaccinate for mpox: cross-sectional web-based survey among sexual and gender minorities. JMIR Public Health Surveill 2023; 9:e46489. 37459174 10.2196/46489 PMC10411424

[10] Oghuan J , Chavarria C, Vanderwal SR, et al Wastewater analysis of mpox virus in a city with low prevalence of mpox disease: an environmental surveillance study. Lancet Reg Health Am 2023; 28:100639. 38076410 10.1016/j.lana.2023.100639 PMC10701415

[11] Telford E , Grinsztejn B, Olsen IC, et al The international unity study for antivirals against mpox is a blueprint for future epidemics. Nat Med 2023; 29:1894–5. 37391664 10.1038/s41591-023-02393-6

[12] Kibungu EM , Vakaniaki EH, Kinganda-Lusamaki E, et al Clade I–associated mpox cases associated with sexual contact, the Democratic Republic of the Congo. Emerg Infect Dis 2024; 30:172–6. 38019211 10.3201/eid3001.231164 PMC10756366

